# Comparing the Efficacy and Safety of the Transperineal Versus Transrectal Prostate Biopsy Approach in the Diagnosis of Prostate Cancer: A Systematic Review and Meta-Analysis

**DOI:** 10.7759/cureus.75459

**Published:** 2024-12-10

**Authors:** Habeeb Abdulrasheed, Althea O George, Petra S Ayobami-Ojo, Pratik Rai, Nwachukwu O Nwachukwu, Aisha Ajimoti, Abdulla Alawadi, Cinzia Z Iftikhar, Aaisha Mehreen, Asante Mbisa

**Affiliations:** 1 Urology, University Hospital Birmingham, Birmingham, GBR; 2 Urology, The Royal London Hospital, London, GBR; 3 Acute Medicine, Princess Alexandra Hospital NHS Trust, Harlow, GBR; 4 Public Health, Liverpool John Moores University, Liverpool, GBR; 5 Nursing, Walsall Healthcare NHS Trust, Walsall, GBR

**Keywords:** cancer detection rate, prostate biopsy complications, prostate cancer (pca), transperineal prostate biopsy, transrectal prostate biopsy

## Abstract

Prostate cancer (PCa) has high prevalence rates in men and is a leading cause of cancer-related death. Transrectal (TR) biopsy has traditionally been the gold standard for diagnosis, but transperineal (TP) biopsy is increasingly favoured due to its lower infection risk. However, debate remains regarding which method has superior cancer detection rates. This review compares the efficacy and safety of the TP as compared to the TR prostate biopsy approach, summarizing the largest body of evidence available to date.

A literature search was performed on the PubMed, Google Scholar, Cochrane Library, and Embase databases. We searched from the inception of the databases up to August 2024 for relevant studies comparing the cancer detection rate of TP versus TR prostate biopsy and compared their complication rates. Twenty-one studies met the inclusion criteria. The pooled odds ratios with 95% confidence intervals were calculated to evaluate the differences between the TR and TP groups in the PCa detection rate.

This meta-analysis included 21 studies (6 randomized control trials and 15 cohort studies) with a total of 13,818 patients (TP = 7917; TR = 5901), who were accrued between 2008 and 2024 and divided into the TR group and the TP group. The analysis revealed no significant difference in prostate cancer detection rates between the TP and TR approaches in both RCTs (OR 1.02, 95% CI (0.74, 1.41), p = 0.90) and cohort studies (OR 1.07, 95% CI (0.85, 1.35), p = 0.36). Complication profiles were largely comparable; TP demonstrated a significantly lower risk of urinary tract infections (OR 0.26, 95% CI (0.11, 0.61)) but no notable differences in acute urinary retention, haematuria, or rectal bleeding.

Our findings advocate the TP approach as a safer biopsy alternative where feasible, particularly in infection-sensitive populations, without compromising diagnostic accuracy. MRI should complement biopsy strategies to enhance diagnostic precision. Future research should focus on standardized, large-scale RCTs to further refine and personalize prostate cancer diagnostic pathways.

## Introduction and background

Prostate cancer (PCa) is the most diagnosed cancer in men, accounting for 29% of all cancer diagnoses, and the second most common cause of cancer deaths in men, after lung cancer [1]. Diagnosis usually follows a routine screening of male adults through rectal examination, prostate-specific antigen (PSA) testing and MRI scan [2]. Prostate biopsy is, however, the only test that is conclusive and dependable in confirming prostate cancer diagnosis and remains the recommended standard of care [3]. Traditionally, transrectal (TR) 12-core sextant biopsy used to be the gold standard and popular approach. However, transperineal (TP) prostate biopsy has emerged as the approach of choice for many, mainly for its reported superiority in terms of cancer detection rate and lower infection risk [4,5].

Prostate biopsy has undergone several revolutions since 1989 when Hodge et al. introduced transrectal-guided biopsy [6]. About three decades later, the PROstate Magnetic resonance Imaging Study (PROMIS) validated the use of multiparametric Magnetic Resonance Imaging (mpMRI) prior to prostate biopsy [7]. Shortly after, the PRostate Evaluation for Clinically Important disease, Sampling using Image-guidance Or Not? (PRECISION) study demonstrated the usefulness of mpMRI-targeted biopsy [8]. Rouviere et al. advocated the benefit of retaining systematic biopsy before Wegelin et al. described the superiority of MRI-TRUS fusion biopsy over cognitive MRI-TRUS and in-bore MRI biopsy [9].

Based on prostate anatomy, two main routes of biopsy have been described. The transrectal route, which is usually an office procedure and is still practised in many countries to date, involves positioning patients in the lateral decubitus position. A digital rectal examination (DRE) is then performed to feel for any nodularity before the ultrasound probe is gently inserted through the rectum. The prostate size is measured before a prostate block is applied by injecting a local anaesthetic in the space between the prostate capsule and the seminal vesicles. Twelve-core sextant biopsies include the peripheral zone bilaterally and the transitional zone in the base, mid and apex of the prostate [6].

The transperineal biopsy is performed in the lithotomy position using the brachytherapy grid and stepper to fix the probe. This technique optimizes access to the anterior lesions, especially in the big glands. At inception, the procedure was carried out under general anaesthesia and patients were at increased risk of urinary retention, bleeding and sepsis. The advent of local anaesthetic transperineal prostate biopsy using deep prostate block has, however, brought about increased acceptability of this route [6].

As the prostate biopsy technique continues to evolve, the desired method of every patient and clinician is to reach the ideal biopsy technique, which has low morbidity and high sensitivity in cancer detection with minimal cost and the best patient experience.

Several studies have been published on the diagnostic approaches of prostate cancer, comparing the transperineal approach to the transrectal. While some found either transperineal or transrectal superior to the other, others found the difference not significant. Our study has gathered up-to-date studies on the comparison of the efficacy and safety of transrectal versus transperineal prostate biopsy. This review, to the best of our knowledge, has the largest number of studies and the largest patient population on this topic.

## Review

Methodology

A systematic review was carried out using the Preferred Reporting Items for Systematic Reviews and Meta-Analyses (PRISMA) checklist. PubMed, Embase, Google Scholar and Cochrane Library were checked for all relevant English language articles. All papers which compared transrectal (TR) and transperineal (TP) prostate biopsies regardless of the year of publication were included. The literature search was performed in August 2024. Mesh words like “Prostate biopsy”, “Transrectal” and “Transperineal” were used. We utilized Boolean operators ‘AND’ and ‘OR’ to refine our search.

Inclusion and exclusion criteria

Studies were considered if they satisfied all the following requirements: (1) they were designed to be an RCT study or cohort study; (2) the subjects of the studies comprised patients who underwent prostate biopsy; (3) the intervention method included the transperineal approach and the transrectal approach; (4) apart from the biopsy approach, the number of cores and the guidance method remained fairly the same; and (5) The outcome of the cases included a diagnosis of prostate cancer (PCa) with or without complications of the two approaches.

Studies whose full content could not be assessed, written in a language other than English, meeting abstracts, and reviews were excluded.

Data Extraction

Data was extracted independently by two authors and all discrepancies were resolved by consultation with a third reviewer. All titles and abstracts found through database searches were independently screened by two reviewers. Rayyan software (https://www.rayyan.ai/) was applied for data processing and duplicate data removal. Following a preliminary title and abstract screening, the chosen papers underwent a full-text review before being accepted or rejected for various reasons. The references of included studies were examined for studies not identified through the original search and included. The data and features of study characteristics (author, publication year, country, study design, sample size), basic patient characteristics (age, PSA, prostate volume, prostate density) and other relevant biopsy details (biopsy technique, PCa detection details, targeted number of cores) were all extracted.

Statistical Analyses

For randomized control trials (RCTs), two independent investigators assessed the risk of bias utilizing the Risk of Bias 2 (RoB 2) tool for randomized trials [10]. For non-randomized studies, the risk of bias was assessed by two investigators independently using the Newcastle Ottawa scale [11].

The studies were RCTs and cohort studies. The odds ratio alongside a 95% confidence interval was generated within a random effect model. If a complication was consistently 0 or not reported within the studies, the complication was excluded from generating an odds ratio. To determine the publication bias, funnel plots were generated to test for asymmetry alongside Egger’s test using a mixed-effects meta-regression model. Standard error was used as the estimator.

Results

Study Characteristics

**Figure 1:**
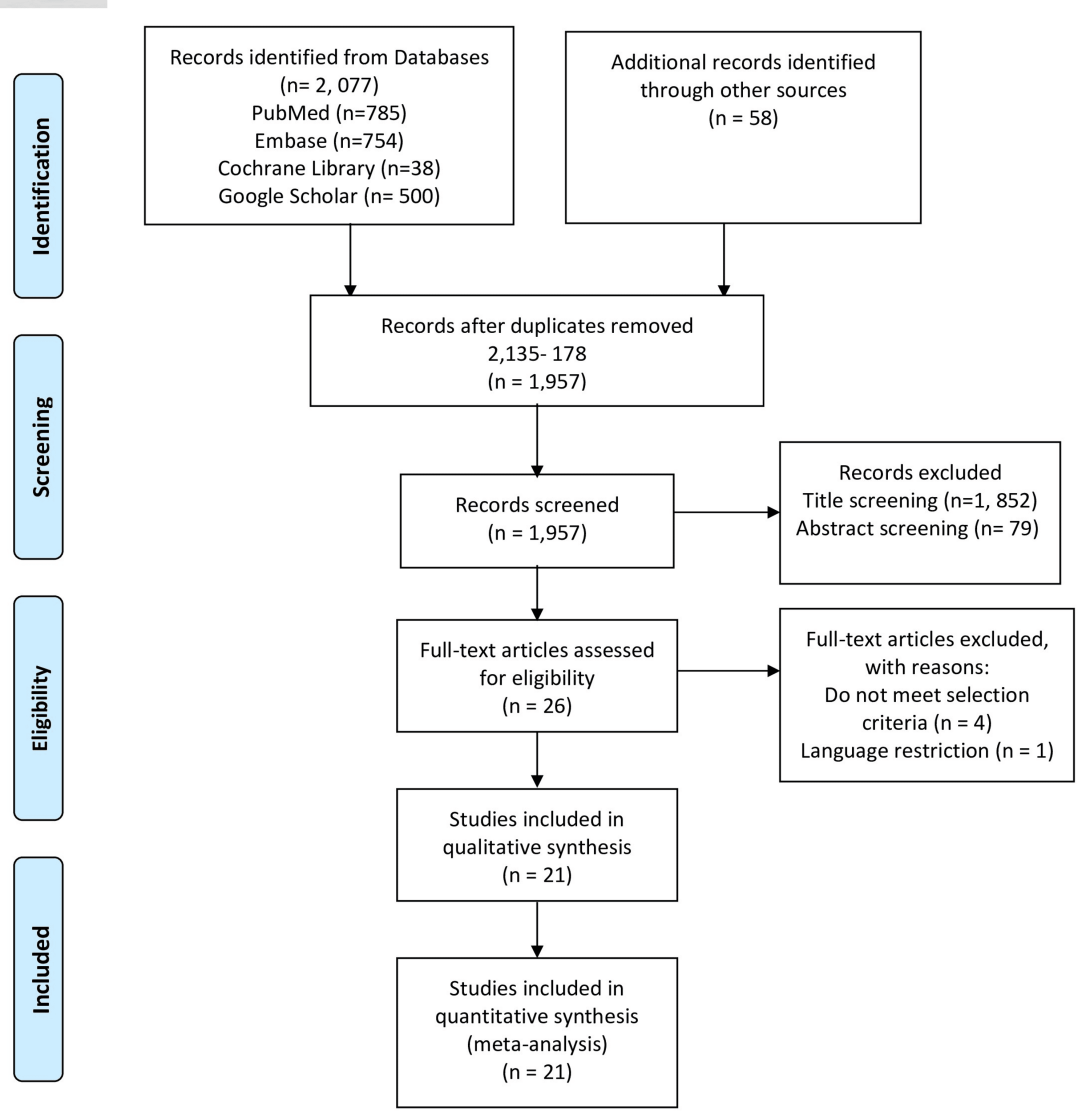
PRISMA flowchart of the studies PRISMA - Preferred Reporting Items for Systematic Reviews and Meta-Analyses, n - Number of articles

A total of 21 studies including 13,818 patients (TP = 7917; TR = 5901) met the inclusion criteria and were involved in the present meta-analysis [12-32]. These studies were carried out between 2007 and 2023. Six of these studies were RCTs and 15 were cohort studies (CS). Figure 1 shows the PRISMA flowchart of the literature search and screening process.

The baseline characteristics of the studies involved are comprehensively listed in Tables 1-2.

**Table 1:** Characteristics of randomized control trials (RCTs) The table provides a summary of the included studies, highlighting their key characteristics, patient demographics and prostate parameters. TP - Transperineal, TR - Transrectal, RCT - Randomised control trials, PSA - Prostate-specific antigen, PV - Prostate volume, PD - Prostate density, DRE - Digital rectal examination, NA - Not available, TRUS - Transrectal ultrasound, MRI - Magnetic resonance imaging, US - Ultrasound, * - Average value

Author	Year	Country	Journal	Design	Biopsy technique	No. of patients	No. of patients		Mean age		Mean PSA		Mean PV		Mean PD		Abnormal DRE		Biopsy cores taken	
							TP	TR	TP	TR	TP	TR	TP	TR	TP	TR	TP	TR	TP	TR
Takenaka et al [12]	2008	Japan	Nature Publishing Group	RCT	TRUS guided	200	100	100	71	72	17.1	19.6	34.5	37.2	0.55	0.61	16	28	12	12
Guo et al [13]	2015	China	Scientific Reports	RCT	TRUS guided	339	173	166	67	67	8.81	10.48	47.2	45.9	N/A	N/A	20	19	12	12
Hara et al [14]	2007	Japan	Ambulatory and Office Urology	RCT	TRUS guided	246	126	120	71	71.7	8.34	8.48	33.2	36	0.3	0.29	14	22	12	12
Rabah et al [15]	2021	Saudi Arabia	Saudi Medical Journal	RCT	MRI/US fusion-guided biopsy TP TR	307	142	165	65	65.1	13.7	14.2	63	61.9	N/A	N/A	14	15	14	14
Cerruto et al [16]	2014	Italy	Archivio Italiano di Urologia e Andrologia	RCT	TRUS guided	108	54	54	66.5	67.3	15.95	12.36	56.29	61.49	NA	NA	11	10	14	14
Ploussard et al [17]	2024	France	European Association of Urology	RCT	MRI-targeted biopsy	250	122	128	66.4	67.3	7.3	7.7	46.7	53.6	NA	N/A	N/A	N/A	12	17
Total/Average*						1450	717	733	67.8*	68.4*	12.5*	12.5*	46.7*	50.0*	0.43*	0.45*	75	94	12.4*	13.5*

**Table 2:** Characteristics of cohort studies The table provides a summary of the included studies, highlighting their key characteristics, patient demographics and prostate parameters. TP - Transperineal, TR - Transrectal, RCT - Randomised control trials, PSA - Prostate-specific antigen, PV - Prostate volume, PD - Prostate density, DRE - Digital rectal examination, NA - Not available, TRUS - Transrectal ultrasound, MRI - Magnetic resonance imaging, US - Ultrasound, * - Average

Author	Year	Country	Journal	Design	No. of patients	Biopsy technique	No. of patients		Mean age		Mean PSA		Mean PV		Mean PD		Abnormal DRE		Biopsy cores taken	
							TP	TR	TP	TR	TP	TR	TP	TR	TP	TR	TP	TR	TP	TR
Chen et al [18]	2021	Singapore	BJU Compass	PCS	372	TRUS-guided	200	172	69	68	13.17	10.76	45.08	49.62	0.29	0.27	102	77	12	12
Winoker et al [19]	2020	USA	Elsevier	PCS	379	MRI-Targeted Software enhanced	168	211	68	65	7.9	7.9	49	51	0.16	0.15	50	52	12	12
Hsiehet al [20]	2022	China	BMC Urology	PCS	177	MR/US software enhanced	92	85	66	67	7.5	8.04	42.34	43.4	N/A	N/A	N/A	N/A	12	12
Kaneko et al [21]	2023	USA	Nature portforlio	PCS	504	MRI/US fusion	168	336	67	66	7.46	7.19	56	52	0.13	0.14	37	108	12	12
Di Franco et al [22]	2017	Italy	Nature Publishing Group	RCS	219	TRUS guided	111	108	68	66	6.9	7.8	N/A	N/A	N/A	N/A	34	42	18	18
Jiang et al [23]	2019	China	Asian Journal of Andrology	RCS	2962	TRUS guided	1746	1216	69.72	69.2	38.02	40.31	51.75	59.64	N/A	N/A	N/A	N/A	12	12
Zattoni et al [24]	2022	Europe	The Journal of Urology	RCS Multicenter	5243	TRUS guided	3,307	1936	68.0	68.3	7.4	7.8	36.1	47.9	0.20	0.17	N/A	N/A	3	4
Lu and Luo et al [25]	2023	China	BMC Urology	RCS	452	TRUS guided	207	245	70.3	70	20.6	23.2	60	54.4	0.47	0.55	N/A	N/A	12	12
Tewes et al [26]	2017	Germany	BioMed Research International	RCS	154	MRI/US fusion Software enhanced	75	79	67	65	16	10	68	58	N/A	N/A	N/A	N/A	12	8
El-Achkar et al [27]	2022	Turkey	Turkish Journal of Urology	RCS	145	MRI/US fusion	98	47	64.5	66	7.5	7.5	51	52.5	0.22	0.19	42	27	11	13
He et al [28]	2022	China	Elsevier	RCS	777	MRI/US fusion software enhanced	540	237	70.5	70.7	14.2	21.9	65.8	62.9	N/A	N/A	N/A	N/A	12	12
Ugge et al [29]	2021	Sweden	Scandinavian Journal of Urology	RCS	105	MRI/US fusion software enhanced	33	72	68	66	6.0	6.7	40	43	16	36	N/A	N/A	5	4
Abdollah et al [30]	2011	Italy	Elsevier	RCS	280	US guided biopsy	140	140	66.4	66.2	10	9.7	62.3	65.4			15	16	24	24
Huang et al [31]	2019	China	BMC Urology	PCS	238	TRUS guided	130	108	66.6	67.1	9.3	10.9	32.5	35	N/A	N/A	N/A	N/A	12	12
Buller et al [32]	2023	USA	Elsevier	RCS	361	MRI targeted	185	176	66	66	6.6	6.4	47.0	55.0	0.13	0.12	N/A	N/A	7	7
Total/ Average*					12368		7200	5168	68.2*	67.7*	17.1*	17.1*	61.4*	57.6*	0.28*	0.30	280	322	11.7*	11.6*

The comparison of study characteristics between TP and TR prostate biopsies across both RCT and cohort studies reveals comparable mean patient ages, PSA levels, prostate volumes, and densities. Abnormal DRE rates prior to biopsy and the number of biopsy cores taken were also similar between the two groups. Tables 1-2 detail these study characteristics, demonstrating that TP and TR have closely aligned baselines, minimized potential bias and confounders and supported robust comparative evaluations of their diagnostic effectiveness.

Prostate Cancer Detection Rate

**Figure 2:**
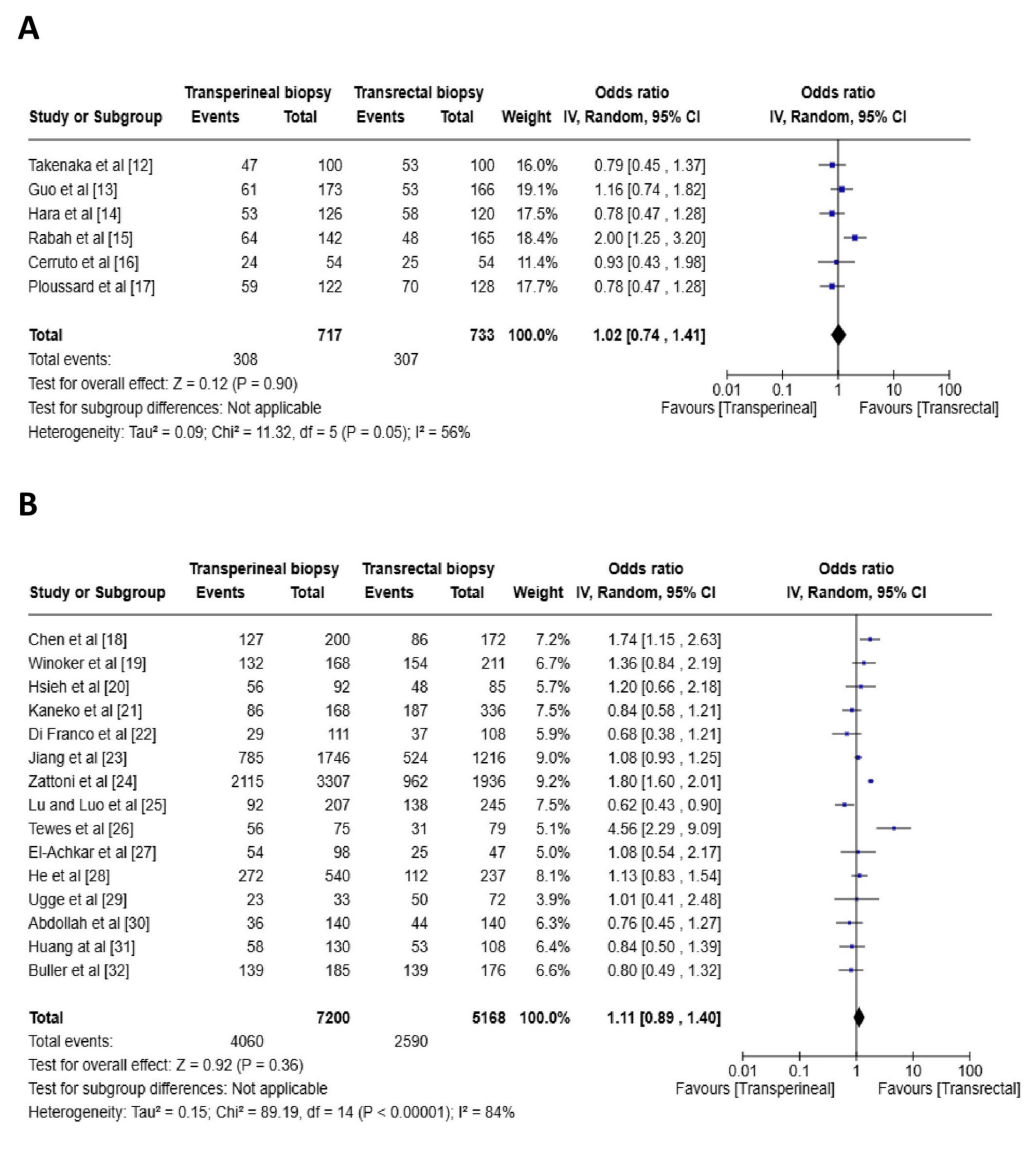
Forest plot for cancer detection rate compared with TP and TR biopsies (A) Forest plot for the cancer detection rate of randomized control trials, (B) Forest plot for the cancer detection rate of cohort studies. The Forest plot compares the efficacy of TP versus TR prostate biopsy in the prostate cancer detection rate. Source: [12-32]

Overall, the RCTs indicated no statistically significant difference in cancer detection rates between the TP and TR prostate biopsy approaches (OR 1.02, CI (0.74, 1.41), p = 0.90). Similarly, the cohort studies also showed no significant difference in cancer detection rates between the two methods (OR 1.11, CI (0.89, 1.40), p = 0.36). Figure 2 shows the forest plots of the studies.

Complications

A summary of the complications from the TP and TR approaches is shown in Table 3.

**Table 3:** Comparing the most common complication rates between the TP and TR biopsy approaches TP - Transperineal, TR - Transrectal, AUR - Acute urine retention, UTI - Urinary tract infection, NA - Data not available

Study	Total Population		Rectal bleeding		AUR		Haematuria		Sepsis		UTI	
	TP	TR	TP	TR	TP	TR	TP	TR	TP	TR	TP	TR
Takenaka et al [12]	100	100	0	1	2	3	11	12	1	2	N/A	N/A
Guo et al [13]	173	166	0	16	N/A	N/A	33	37	2	10	N/A	N/A
Hara et al [14]	126	120	0	0	2	3	13	11	0	2	N/A	N/A
Rabah et al [15]	142	165	0	6	8	7	1	2	N/A	N/A	0	0
Cerruto et al [16]	54	54	0	4	NA	N/A	5	0	0	1	NA	N/A
Ploussard et al [17]	122	128	N/A	N/A	N/A	N/A	N/A	N/A	N/A	N/A	N/A	N/A
Chen et al [18]	200	172	N/A	N/A	8	8	2	3	0	4	2	4
Winoker et al [19]	168	211	N/A	N/A	6	1	N/A	N/A	0	1	N/A	N/A
Hsieh et al [20]	92	85	3	3	17	4	11	17	0	1	1	1
Kaneko et al [21]	168	336	0	0	1	3	N/A	N/A	N/A	N/A	0	4
Di Franco et al [22]	111	108	0	4	2	3	3	3	N/A	N/A	0	3
Jiang et al [23]	1746	1216	N/A	N/A	N/A	N/A	N/A	N/A	N/A	N/A	N/A	N/A
Zattoni et al [24]	3,307	1936	N/A	N/A	N/A	N/A	N/A	N/A	N/A	N/A	N/A	N/A
Lu and Luo et al [25]	207	245	N/A	N/A	N/A	N/A	N/A	N/A	N/A	N/A	N/A	N/A
Tewes et al [26]	75	79	N/A	N/A	N/A	N/A	N/A	N/A	N/A	N/A	N/A	N/A
El-Achkar et al [27]	98	47	N/A	N/A	0	1	0	1	1	2	1	5
He et al [28]	540	237	NA	N/A	27	15	NA	N/A	10	16	6	8
Ugge et al [29]	33	72	N/A	N/A	N/A	N/A	N/A	N/A	N/A	N/A	NA	N/A
Abdollah et al [30]	140	140	N/A	N/A	N/A	N/A	N/A	N/A	N/A	N/A	N/A	N/A
Huang et al [31]	130	108	2	0	4	9	7	15	1	20	3	13
Buller et al [32]	185	176	N/A	N/A	5	3	2	1	1	3	N/A	N/A
Total	7917	5901	5	34	80	62	88	102	16	62	13	38

The RCTs showed no significant difference in acute urinary retention (AUR) between the TP and TR biopsy approaches (OR 1.00, CI (0.45, 2.25)). Similarly, no significant difference was observed in haematuria (OR 0.93, CI (0.63, 1.38)). Rectal bleeding also did not significantly differ between the two approaches (OR 0.19, 95% CI (0.03, 1.02)).

In cohort studies, there was no significant difference in AUR (OR 1.11, 95% CI (0.50, 2.47)) or haematuria (OR 0.52, 95% CI (0.3, 0.9)). However, cohort studies indicated a lower risk of urinary tract infections (UTI) with the TP approach compared to the TR approach (OR 0.26, 95% CI (0.11, 0.61)).

Test of Heterogeneity

**Figure 3:**
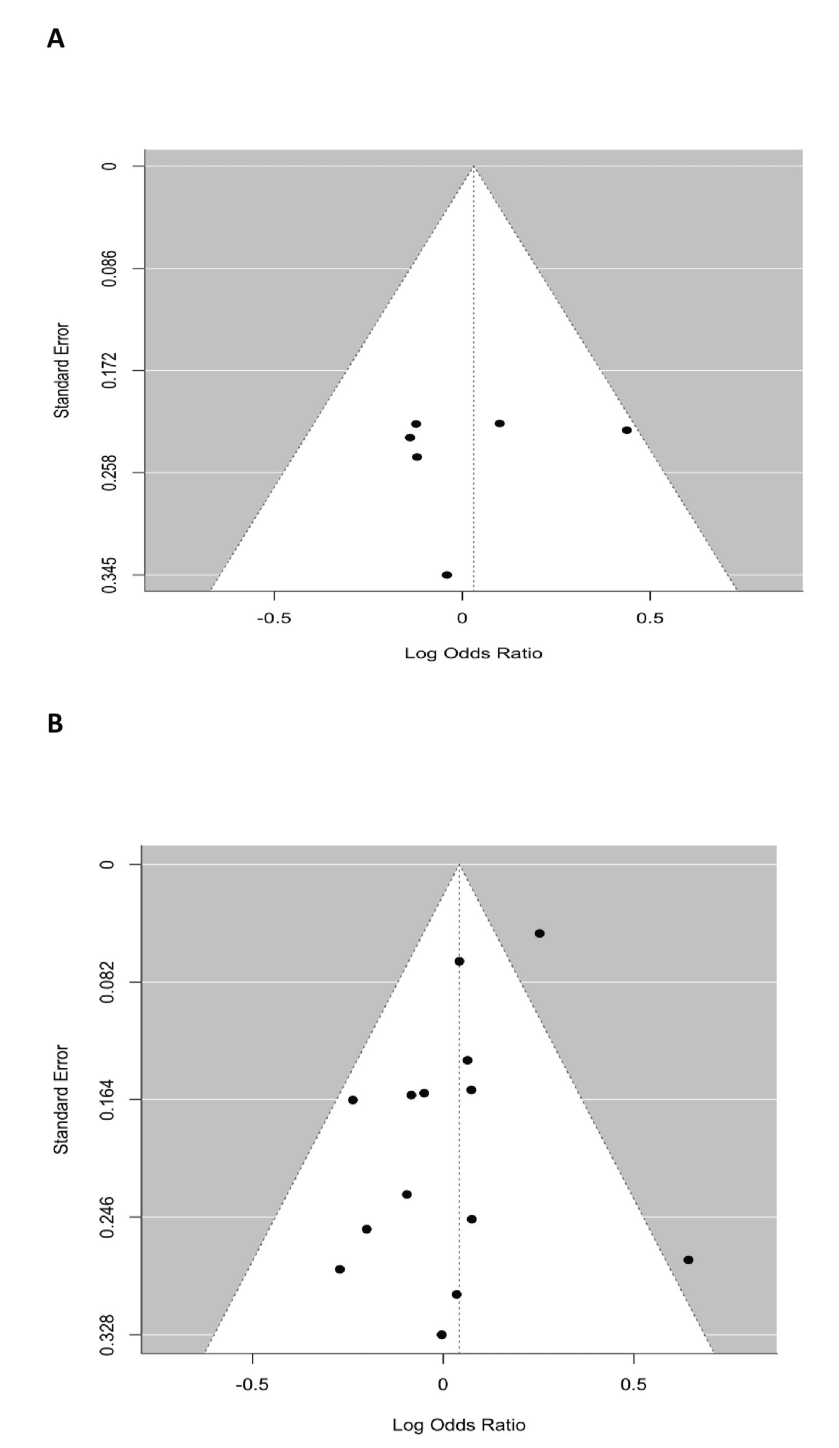
Funnel plots of asymmetry of the studies (A) Funnel plot asymmetry for RCTs (z = -0.3771, p = 0.7061), (B) Funnel plot asymmetry for cohort studies (z = -1.3312, p = 0.1831) RCT - Randomized controlled trials

The funnel plot of our study is symmetric, indicating no significant evidence of publication bias in the meta-analysis comparing prostate cancer detection rates between the TP and TR biopsy approaches (z = 0.0093, p = 0.9926). The limit estimate as the standard error approaches zero (b = -0.0062, CI: -0.8134 to 0.8009) further supports the absence of asymmetry. These results suggest that the findings of this meta-analysis are robust and not influenced by publication bias, supporting the reliability of our comparison of prostate cancer detection rates between the TP and TR approaches. Figure 3 shows the funnel plots for both RCTs and cohort studies.

Publication Bias

**Figure 4:**
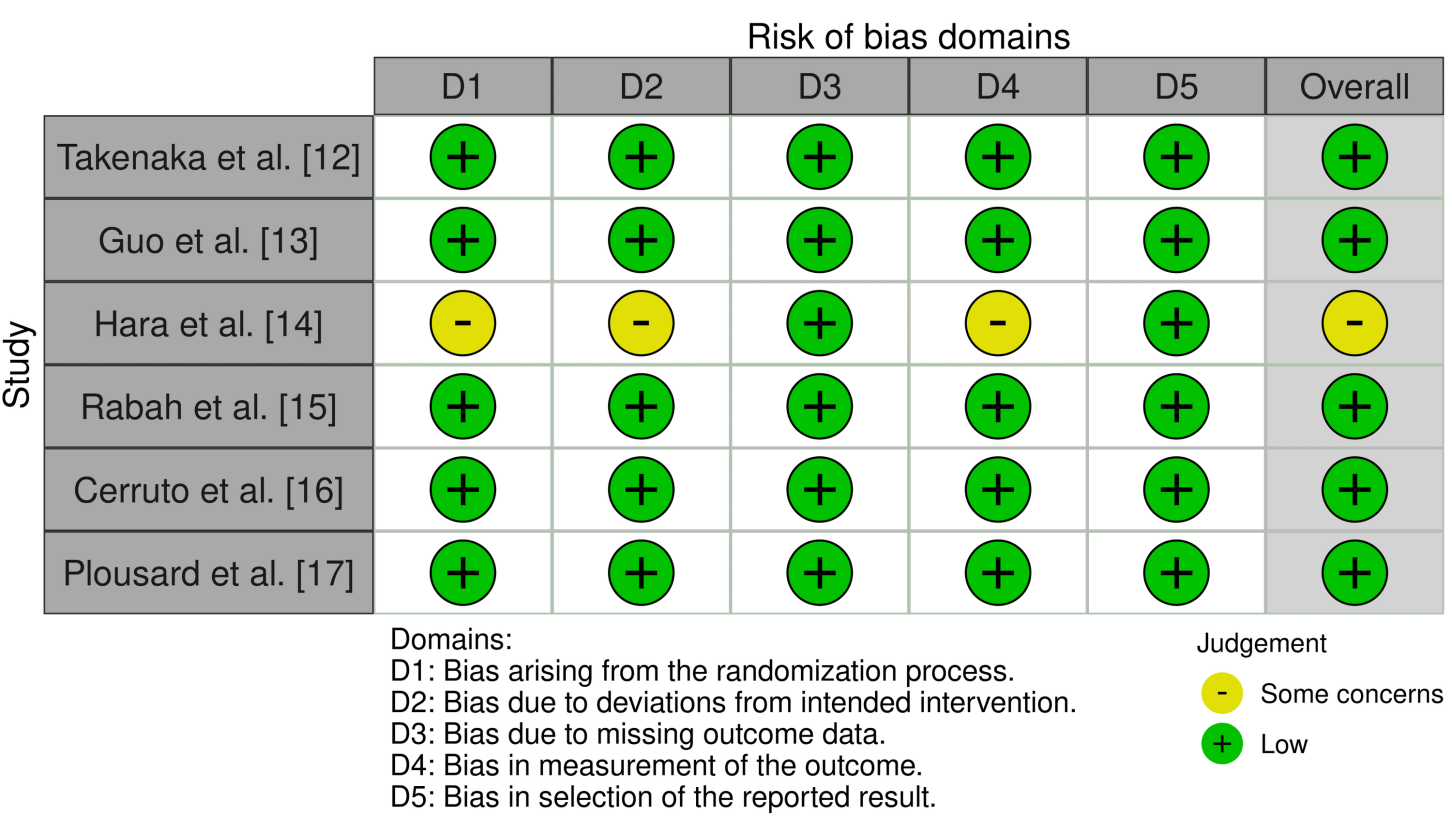
Risk of bias of the included randomized control trials

In Egger’s test for the cancer detection rate, both RCTs (p = 0.7) and cohort studies (p = 0.18) did not demonstrate significant asymmetry and thus did not show a significant publication bias. Similarly, Egger’s test for AUR with RCTs (p = 0.9) and cohort studies (p = 0.61) did not display a significant publication bias. The risk of bias for both RCT and cohort studies is shown in Figure 4 and Table 4, respectively.

**Table 4:** Risk of bias of the included cohort studies ✓ (met criteria), ✗ (criteria unmet); Scoring (<4 = high risk, 4–6 = moderate, and ≥7= low risk)

Author	Selection				Comparability of cohorts based on design and analysis (0, 1, 2 points)	Outcome			Total (max=9 points)	Risk of bias
	Representativeness of exposed cohort	Selection of non-exposed cohort	Ascertainment of exposure	Outcome present at the start of the study		Assessment Of outcome	Follow-up sufficient for outcome To occur	Adequate follow-up of cohorts		
Chen et al [18]	✓	✓	✓	✓	2	✓	✓	✓	9	low
Winoker et al [19]	✓	✓	✓	✓	1	✓	✓	✓	8	low
Hsiehet al [20]	✓	✓	✓	✓	2	✓	✓	✓	9	low
Kaneko et al [21]	✓	✓	✓	✓	1	✓	✓	✓	8	low
Di Franco et al [22]	✓	✓	✓	✓	1	✓	✓	✓	8	low
Jiang et al [23]	✓	✓	✓	✓	1	✓	✓	✓	8	low
Zattoni et al [24]	✓	✓	✓	✓	2	✓	✓	✓	9	low
Lu and Luo et al [25]	✓	✓	✓	✓	2	✓	✓	✓	9	low
Tewes et al [26]	✓	✓	✓	✓	1	✓	✓	✓	8	low
El-Achkar et al [27]	✓	✓	✓	✓	1	✓	✓	✓	8	low
He et al [28]	✓	✓	✓	✓	1	✓	✓	✓	8	low
Ugge et al [29]	✓	✓	✓	✓	1	✓	✓	✓	8	Low
Abdollah et al [30]	✓	✓	✓	✓	1	✓	✓	✓	8	low
Huang et al [31]	✗	✗	✓	✓	1	✓	✓	✓	6	moderate
Buller et al [32]	✓	✓	✓	✓	2	✓	✓	✓	9	Low

Discussion

This meta-analysis, comprising 6 RCTs [12-17] and 15 cohort studies [18-32], demonstrates no significant difference in prostate cancer detection rates between the TP and TR biopsy approaches. Although TP biopsy exhibits a better safety profile, our findings indicate that most reviewed complications do not differ significantly between the two methods.

Cancer Detection Rate

Our meta-analysis found no significant difference in prostate cancer detection rates between the TP and TR biopsy approaches, with pooled estimates from RCTs (OR 1.02, CI (0.74, 1.41), p = 0.90) and cohort studies (OR 1.11, CI (0.89, 1.40), p = 0.36) supporting this conclusion. These findings align with previous research conducted over the years. For example, Shen et al., in a systematic review of three RCTs and four case-control studies, found no significant difference in cancer detection rates between the TP and TR biopsy techniques [33]. Xue et al. also reported no significant differences in detection rates between the two approaches [34].

Furthermore, Xiang et al. conducted a systematic review of four RCTs and seven cohort studies and similarly found no significant difference in detection rates (RR 0.94, 95% CI (0.81, 1.10), p = 0.678) [35]. More recently, Wu et al. reported that while TP detected more prostate cancer overall (RR 1.25, CI (1.12, 1.39), p < 0.0001), there was no significant difference in the detection of clinically significant cancer between the TP and TR approaches (RR 0.91, CI (0.76, 1.08), p = 0.28) [36]. The most recent study by Zattoni et al., comprising three prospective RCTs comparing TP versus TR MRI-targeted biopsies, observed no significant variation in detection rates (OR 0.9, CI (0.7, 1.1)) [37]. These studies, taken together, reinforce the conclusion that both TP and TR biopsy approaches yield comparable prostate cancer detection rates.

Complications

While TP generally has a better safety profile due to its reduced risk of infection, specific complications analyzed in our study show no statistically significant differences between the two approaches.

Bleeding Complications

Rectal bleeding:* *TP minimizes rectal bleeding, with significantly fewer cases reported (5 cases in TP vs. 34 in TR). However, an RCT analysis showed no significant difference in rectal bleeding between the two approaches (OR 0.19, CI (0.03, 1.02)). This result contrasts with findings from Xiang et al., who reported that TP significantly reduced the risk of rectal bleeding. Since TR biopsy is performed through the rectal route, while TP biopsy is conducted via the perineum, it is not surprising that TR carries a higher risk of rectal bleeding as compared to TP [35].

Haematuria: Our meta-analysis showed no significant difference in haematuria rates between the TP and TR approaches (OR 0.93, CI (0.63, 1.38)) and this aligns with previous similar research. Miller et al. reported comparable haematuria rates of 14.8% for TP and 16.4% for TR (p = 0.766) [38], and Xiang et al. found similar results with a relative risk of 0.79 (CI (0.63, 1.01)) [35].

Non-bleeding Complications

Acute urine retention (AUR): Despite more reported cases of AUR in TP (80 vs. 59 cases across 12 studies), a meta-analysis showed no significant difference in AUR rates between TP and TR with the pooled results from RCTs (OR 1.00, CI (0.45, 2.25)) and cohort studies (OR 1.11, CI (0.5, 2.47)) supporting this conclusion.

These results are consistent with those of Xiang et al., who found no significant difference, with 20 of 806 patients in the TP group and 25 of 812 in the TR group reporting AUR [35]. Similarly, Chen et al. reported comparable AUR rates of 3.8% for the TP group and 4.5% for the TR group (p = 0.8) [20]. Zattoni et al. also found similar rates of AUR, with 0.5% in the TP group and 1.0% in the TR group [37].

Sepsis: TP significantly reduces the risk of sepsis as the needle bypasses the rectal mucosa. While our study reported lower sepsis rates in TP (1.2%) compared to TR (4.9%), some studies found no difference. Chen et al. reported no cases of urosepsis in the TP group as compared to 2.2% in the TR group (p = 0.04) [20]. On the other hand, Miller et al. found a higher rate of sepsis in the TP group (1.2%) compared to TR (0%) although the difference is not significant (p = 0.411) [38].

Strengths and limitations of the studies

Our study is the most comprehensive meta-analysis to date, utilizing high-quality RCTs and cohort studies (NOS score >6) and adhering to PRISMA guidelines. By separately analyzing RCTs and cohort studies, we minimized methodological biases.

However, limitations include the small number of RCTs, which are considered the gold standard in clinical trials. Additionally, potential confounders, such as prostate size, PSA levels, number of cores taken, and patient health background, may influence the findings. These limitations highlight the need for more large-scale, standardized RCTs to validate our conclusions.

## Conclusions

The transperineal (TP) and transrectal (TR) prostate biopsy approaches demonstrate comparable diagnostic accuracy for prostate cancer detection. However, the TP approach offers a superior safety profile, notably reducing infection risks and rectal bleeding. As such, TP is recommended where feasible, especially in clinical settings prioritizing safety and infection control. Incorporating MRI-guided biopsies, as validated by the PROMIS and PRECISION trials, can further enhance diagnostic precision and reduce unnecessary interventions. Future research should focus on large-scale, high-quality randomized controlled trials to refine and optimize biopsy strategies, ensuring improved outcomes for prostate cancer diagnosis.
